# Factors influencing delay in malaria treatment seeking at selected public health facilities in South Gonder, Ethiopia

**DOI:** 10.1038/s41598-024-56413-7

**Published:** 2024-03-20

**Authors:** Adimasu Alga, Yitbarek Wasihun, Tiruneh Ayele, Abel Endawkie, Sefineh Fenta Feleke, Natnael Kebede

**Affiliations:** 1 Department of Reproductive and Family Health, School of Public Health, College of Medicine Health Sciences, Wollo University, Dessie, Ethiopia; 2 Department of Health Promotion, School of Public Health, College of Medicine Health Sciences, Wollo University, Dessie, Ethiopia; 3 Department of Epidemiology and Biostatics, School of Public Health, College of Medicine Health Sciences, Wollo University, Dessie, Ethiopia; 4 Department of Public Health, Woldia University, Woldia, Ethiopia

**Keywords:** Determinants, Treatment seeking, Malaria patients, Ethiopia, Diseases, Health care

## Abstract

Early and prompt treatment-seeking for malaria is necessary to reduce the progression of the disease to its severe forms and the associated mortality. Various studies have indicated that treatments sought for malaria were not always within the recommended timeframe. Therefore, this study aims to assess factors influencing delay in malaria treatment seeking at public health facilities in South Gonder, Ethiopia. An unmatched case–control study was conducted among 322 individuals, comprising 161 cases and 161 controls, who were randomly selected malaria patients visiting public health facilities in South Gonder District, Ethiopia, from May 20/2022 to June 25/2022. An interviewer-administered questionnaire was used to collect data, which were subsequently cleaned and entered into Epi data. Descriptive statistics were performed, and variables with a p-value of ≤ 0.25 from the bivariate analysis were included in a multivariable logistic regression model. Significant variables with a p-value of < 0.05 were retained in the multivariable model. Patients who were unable to read and write [AOR = 3.47 (1.01–11.9)], fear of side effects of malaria treatment drugs [AOR = 1.89 (1.04–3.42)], lack of access to health education malaria disease and its treatment [AOR = 1.93 (1.02–3.65)], lack of transportation access [AOR = 4.70 (1.73–12.7)], not membership of community-based health insurance [AOR = 2.5 (1.3–4.82)] and lack of confidence on malaria care health facility providing [AOR = 2.14 (1.06–4.29)], were found to be determinants of treatment-seeking delay among malaria patients. In Summary, this study revealed significant associations between delays in seeking malaria treatment and factors such as educational status (those who were unable to read and write), malaria drug side effects, health education on malaria, transportation access, CBHI membership, and confidence in health malaria care. it is recommended that targeted interventions and awareness campaigns be implemented to address these determinants, promoting prompt and effective malaria treatment-seeking behavior in the studied population.

## Introduction

Malaria, a communicable disease caused by the Plasmodium parasite and transmitted through Anopheles mosquitoes, is a significant global health concern^1,2^. The World Health Organization (WHO) emphasizes key pillars for effective malaria prevention and control, with a crucial focus on ensuring universal access to prevention, diagnosis, and treatment^3,4^. The impact of malaria is particularly pronounced in sub-Saharan Africa, where 90% of all malaria-related deaths occur, making it a leading cause of morbidity and mortality worldwide^3,5^. Early and appropriate treatment plays a pivotal role in preventing the progression of malaria to severe stages, ultimately reducing morbidity and mortality while limiting further transmission^6^. An additional objective of early diagnosis and treatment is to diminish the reservoir of individuals carrying the malaria parasite, thereby lowering the risk of transmission within the community. Recognizing these critical aspects, it is recommended that individuals gain access to early diagnosis and receive prompt, effective treatment within 24 h of experiencing malaria symptoms^7,8^.

Different studies have revealed that delay in treatment-seeking is a problem, and its burden is higher in regions with a higher malaria prevalence. The East Asian region is one such area with a high malaria burden, where the delay in treatment-seeking is surprisingly 100%. The lowest percentage of delay experienced in this region was 79.4%. In the African continent delay in treatment-seeking for malaria ranges from 36.6 to 87.8%^8^. In Ethiopia in particular, delay in treatment-seeking for malaria ranges from 28.7 to 52.4%, which is still far from the country's target of zero percent delay^8–11^.

The National Malaria Control Programme(NMCP) 2017–2020 which Ethiopia is implementing specifies that 100% of suspected malaria cases should be diagnosed within 24 h of the onset of symptoms^12,13^. However, contrary to this ambitious goal, studies conducted in some parts of the country revealed that the percentage of patients seeking treatment within the recommended 24 h of onset of symptoms is very low^9,14,15^. Even among the findings of studies in Ethiopia, the maximum percentage of malaria patients who sought treatment within 24 h of the onset of symptoms is 52.4%, which is only half of Ethiopia's national target^16,17^.

Socio-demographic characteristics, physical accessibility & environmental factors, experience & behavioral factors, and knowledge about malaria were reported as factors affecting treatment-seeking in different studies^15,17^. Several behavioral aspects contribute to this delay, including a lack of knowledge about malaria symptoms, belief in traditional remedies, fear of negative side effects of medication, financial constraints, and distance to health facilities. Patients may also be deterred by stigma associated with seeking medical care, perception of mild illness, and perceived low quality of care in public facilities^18,19^.

In Ethiopia, most of the studies about factors associated with delay in treatment-seeking were confined to under-five children^9,20^. Even though children of age less than 5 years are in the high-risk group for malaria, it is also important to consider the older population because, according to Ethiopia’s 2019 malaria report, during the last 6 years numbers have shown that 80% of malaria victims were adults and children at least 5 years of age^21^.

Additionally, the South Gonder District is an area where malaria is prevalent. Understanding the factors that contribute to delays in seeking treatment among malaria patients in this district can provide valuable insights for health program planners, NGOs, researchers, and other organizations dedicated to reducing the impact of malaria on mortality and morbidity. While previous studies have explored various aspects of malaria prevention and control, there is a noticeable lack of comprehensive investigations into the specific factors that contribute to delays in seeking treatment at public health facilities in the South Gonder region. The study addresses crucial gaps in understanding factors contributing to delays in malaria treatment seeking, offering valuable insights for policymakers, partners, and implementers in malaria prevention, control, and elimination efforts, Therefore, this study aimed to assess factors influencing delay in malaria treatment seeking at public health facilities in South Gonder, Ethiopia.

## Methods and materials

### Study design and period

A facility-based, unmatched case–control study was carried out, involving 322 randomly selected malaria patients (161 cases and 161 controls) in public health facilities in South Gonder District, Ethiopia from May 20/2022 to June 25/2022. South Gonder District is situated in the Amhara region, in the north-central part of Ethiopia, positioned between 11°8' N and 39°38' E latitude and 11.133° N 20' and 39.633° E longitude. The district spans an elevation range from 2470 to 16,840 m above sea level, covering a surface area of approximately 16,840 km^2^. In terms of geographical proximity, South Gonder District is located 666 km away from Addis Ababa, the capital city of Ethiopia, and 97 km from Bahir Dar, the capital city of the Amhara Region.

### Population

All confirmed malaria patients who seek treatment in public health facilities of South Gonder District were taken as the source population. All confirmed malaria patients who sought treatment in public health facilities of South Gonder District during the data collection period were considered as the study population.

Cases: Identified as a malaria patient who seeks treatment after 24 h of the onset of the first symptoms of malaria^22^.

Controls: Identified as a malaria patient who seeks treatment within 24 h of onset of the first symptom of malaria^23^.

### Inclusion and exclusion criteria

All confirmed malaria patients for *Plasmodium* species by blood film (Bf) or rapid diagnostic test (RDT) enrolled in the study were included in the study. While those who were critically ill were excluded from the study.

### Sampling method and sample size determination

The sample size was determined by using the double population proportion formula for the case–control study in Epi-info 7.0 stat calc. The calculation was computed by taking three variables; the presence of side effects, knowledge of malaria, and distance of the health facility which are significantly associated with treatment-seeking delay in most studies. The final sample size was calculated by using the assumption of "presence of side effect" with the odds ratio of 4.96, the percent of controls exposed at 2.6%, the percent of cases exposed at 11.7% with a 95% confidence interval, power being 80%, case to control the ratio of 1:1 and by adding 10% nonresponse rate^20^. The total number of malaria patients was 17,622 from public health facilities. The study's ultimate sample comprised 322 participants, evenly divided into 161 cases and 161 controls at a one-to-one ratio. Both cases and controls were chosen separately through a systematic random sampling method. Cases were initially selected using a lottery, and subsequently, every fourth interval was chosen, as illustrated below. (Table 1).

**Table 1:** Variables used to calculate the largest possible sample size.

Variables	CI	Power (%)	% of controls exposed (%)	% cases exposed (%)	Cases to controls ratio	Odds ratio (OR)	Total sample size
Presence of side effects	95%	80	2.6	11.7	1:1	4.96	322
Knowledge of malaria	95%	80	34.2	51.2	1:1	2.02	286
Distance of the health facility	95%	80	35.5	52.5	1:1	2.01	288

Ninety-six public health facilities exist in South Gonder District, and for participant selection, nine were chosen randomly through a lottery method. This approach ensured an equal opportunity for all facilities, maintaining a fair and unbiased selection process. Cases were included by using a systematic random sampling technique by calculating the sampling interval for cases based on the average monthly cash flow of each health facility while controls were selected as cases were enrolled.

The Kth (K = 4) value was determined for both cases and controls; it was done separately by dividing the patient flow by sample size (the total malaria patient flow of South Gonder District of month June 2013 E.C was 1288 from the data that was collected from each facility. The study subject was selected by using systematic random sampling techniques after a laboratory request was done and when patients those positive for any species of *Plasmodium*, they were eligible and were moved to a private room for an interview until the total required sample size was obtained.

### Operational definitions

#### Knowledge about malaria

Assessed by ten knowledge assessment questions and those who scored 70% and more were considered as having adequate knowledge and those who scored less than 70% on knowledge assessment questions were considered as having inadequate knowledge^24^.

#### Timely treatment-seeking

This is a treatment sought for symptoms of malaria within 24 h of the onset of symptoms^23,25^.

#### Delayed treatment-seeking

This is a treatment sought after the recommended time of 24 h onset of malaria symptoms^23,25^.

#### Waiting time

The time a patient had to wait at registration, consultation, laboratory, and other diagnostic units and at the pharmacy to receive service. If the patient's waiting time is greater or equal to 120 min considered a long waiting time and if the waiting time is less than 120 min it is considered a short waiting time^22^**.**

#### Client satisfaction

A subjective measure of whether a patient’s expectations about a health encounter were met^22^.

#### Confidence in healthcare

Confidence in the healthcare system implies an expectation that sufficient and appropriate treatments will be provided if needed^23^.

### Data collection procedure and quality assurance

The questionnaire was initially developed in English by reviewing available literature. The training was given to data collectors and a supervisor. Data were collected by a structured pre-tested questionnaire which was developed by reviewing different peer-reviewed literature^26,27^. The tool consists of sociodemographic characteristics, physical accessibility and environmental factors, behavioral factors, and questions on treatment-seeking delay. Additionally, knowledge assessment questions on signs/symptoms, transmission, and prevention of malaria were partially adapted from a previously conducted study which contains 12 closed-ended and multiple choice questions where each choice has a score of 1 point^27,28^.

### Data processing and analysis

After ensuring completeness and consistency, the data underwent coding and entry into EPI-data v 4.6.0.2, followed by exportation to SPSS 23 statistical software for processing and analysis. Frequency tables and descriptive summaries were employed to depict study variables. Logistic regression analysis assessed the significance of associations between dependent and independent variables. Bivariate and multivariable analyses were conducted to examine the relationship between treatment-seeking delay among malaria patients and selected independent variables. In bivariate logistic regression, variables with a p-value of 0.25 or less were included in the multivariable logistic regression analysis^29^, then p value less than 0.05 was considered a predictor for the delay in treatment-seeking among malaria, and an adjusted odds ratio with 95% CI was considered to see the strength and significance of the association. The adequacy of the final model was checked using the Hosmer and Lemeshow Goodness of fit test. With p- a value was 0.08^30^.

### Ethical approval and consent to participate

Ethical approval was obtained from the Institutional Review Board of Wollo University, College of Medicine and Health Science with the reference number of Ref no: CMHS/85/13/2022. Following the approval, an Official letter of cooperation was written to concerned bodies by the School of Public Health of Wollo University. Permission was also obtained from the South Gonder District health department and respective health facilities in which the data collection was conducted. Respondents were informed about the objective and purpose of the study and verbal informed consent was obtained from each respondent while permission from parents or guardians was obtained for participants of age less than 18 years also permission was taken from the participants for ages greater and equal to 18 years. Clear information was given to inform respondents about the purpose and procedure of the study, the importance of their participation, and the right to withdraw at any time if they want. Additionally, the respondents were informed about the privacy and confidentiality of the information they provided throughout the study, by maintaining anonymity and interviewing them in a separate room during the interview. All methods were carried out under relevant guidelines and regulations.

## Results

### Socio-demographic characteristics of participants

A total of 322 study participants (161 cases and 161 controls) were successfully involved in the study. Among the cases (N = 161), 58.4% were male, with 90.1% residing in rural areas. The majority belonged to the Amhara ethnicity (87%) and were married (57.8%). Regarding education, 37.9% were unable to read and write. In terms of occupation, 23.6% were students, and 21.1% were farmers. Monthly income revealed that 72% fell into the lower-income category. Family size varied, with 79.5% having five or more members. Controls (N = 161) exhibited similar trends, with 64.6% being male, 83.2% residing in rural areas, and 81.4% belonging to the Amhara ethnicity. The majority were married (59.6%), and 34.2% had a college education or above (Table 2).

**Table 2:** Socio-demographic characteristics of study participants in public health facilities of South Gonder District, Ethiopia, 2022(n = 322).

	Patient categories (N = 322)	
	Cases (N = 161)	Controls (N = 161)
Variables		
Sex		
Male	94 (58.4%)	104 (64.6%)
Female	67 (41.6%)	57 (35.4%)
Place of residence		
Urban	16 (9.9%)	27 (16.8%)
Rural	145 (90.1%)	134 (83.2%)
Ethnicity		
Amhara	140 (87%)	131 (81.4%)
Oromo	8 (4.9%)	9 (5.6%)
SNNP	5 (3.1%)	7 (4.3%)
Other	8 (4.9%))	14 (8.7%)
Marital status		
Single	45 (28%)	46 (28.6%)
Married	93 (57.8%)	96 (59.6%)
Widowed	12 (7.5%)	12 (7.5%)
Divorced	11 (6.8%)	7 (4.3%)
Religion		
Orthodox	108 (67.1%)	122 (75.8%)
Muslim	34 (21.1%)	25 (15.5%)
Other	19 (11.8%)	14 (8.7%)
Educational status		
Unable to read and write	61 (37.9%)	30 (18.6%)
Primary school	48 (29.8%)	42 (26.1%)
Secondary school	28 (17.4%)	34 (21.1%)
College and above	24 (14.9%)	55 (34.2%)
Occupation		
Government employee	13 (8.1%)	38 (23.6%)
Private employee	29 (18%)	29 (18%)
Housewife	22 (13.7%)	27 (16.8%)
Student	38 (23.6%)	18 (11.2%)
Farmer	34 (21.1%)	23 (14.3%)
Daily labourer	13 (8.1%)	10 (6.2%)
Merchant	12 (7.5%)	16 (9.9%)
Monthly income		
Lower income (below 3084 ETB)	116 (72%)	85 (52.8%)
Higher income (above 3084 ETB)	45 (28%)	76 (47.2%)
Family size		
Less than five	33 (20.5%)	80 (49.7%)
Five or more	128 (79.5%)	81 (50.3%)

### Duration of treatment-seeking and Reasons for delay in treatment-seeking for malaria among cases

Out of the 161 cases, a significant majority, comprising 132 individuals (81.9%), sought medical attention at a health facility within 24 to 48 h of experiencing the initial signs and symptoms. A notable proportion (56.5%) attributed their delayed treatment seeking for malaria to challenges such as limited access to transportation and the elevated cost of medical care associated with seeking treatment at a health facility.

### Knowledge about malaria

In this study, 97 (60.2%) of the controls and 71 (44%) of the cases had adequate knowledge about malaria. The majority of the cases (95%) and controls (92.5%) had heard about malaria. Most of the controls 118 (80.3%) and cases 115 (75.2%) had known mosquito bite causes malaria. The majority of the controls (66.4%) and almost half of the cases (54.2%) perceived malaria as a serious and killer disease. Almost all of the controls (95.8%) had listed two or more malaria symptoms. Most of the controls (85.8%) and half of the cases (56.7%) had known how to prevent malaria (Table 3).

**Table 3:** Knowledge about malaria among study participants in public health facilities of South Gonder District, 2022(n = 322).

Knowledge questions		Patient categories (N = 322)	
		Cases (N = 161)	Controls (N = 161)
Ever heard about malaria	No	8 (5%)	12 (7.5%)
	Yes	153 (95%)	149 (92.5%)
Malaria caused by mosquito bite	No	38 (24.8%)	43 (26.8%)
	Yes	123 (75.2%)	118 (73.2%)
Malaria can be prevented	No	52 (32.3%)	35 (14.3%)
	Yes	109 (67.7%)	126 (85.7%)
Malaria can be cured	No	24 (14.9%)	24 (14.9%)
	Yes	137 (85.1%)	137 (85.1%)
Malaria can cause death	No	78 (48.5%)	66 (33.6%)
	Yes	83 (51.5%)	95 (66.4%)
Had listed two or more symptoms of malaria	No	26 (16.2%)	18 (11.2%)
	Yes	135 (83.8%)	143 (88.8)
Had known mosquito biting time	Incorrect	85 (51%)	78 (48.4%)
	Correct	76 (49%)	83 (51.6%)
Had known the resting places of mosquitoes	No	119 (73.9%)	55 (34.2%)
	Yes	42 (26.1%)	106 (65.8%)
Had known the breeding sites of mosquitoes	No	68 (42.2%)	26 (16.1%)
	Yes	93 (57.8%)	135 (83.9%)
Had known how to prevent mosquito breeding site	No	72 (44.7%)	22 (13.7%)
	Yes	89 (55.3%)	139 (86.3%)

### Behavioural factors affecting treatment seeking for malaria

The study on behavioral factors influencing malaria treatment-seeking behavior in South Gonder District, Ethiopia (N = 322), reveals distinctive patterns among cases (N = 161) and controls (N = 161). Perception of medication side effects varied, with 60.9% of cases and 39.8% of controls expressing concerns. Health facilities were the primary choice for seeking treatment, particularly for cases (70.8%). Traditional medicines were utilized by 27.3% of cases and 25.5% of controls upon malaria symptoms onset. Moreover, self-medication was practiced by 28.6% of cases and 23.6% of controls. The decision-maker for seeking treatment predominantly was the patient, representing 62.1% of cases and 69.6% of controls(Table 4).

**Table 4:** Behavioural factors affecting treatment-seeking for malaria among study participants in public health facilities of South Gonder District, Ethiopia, 2022 (n = 322).

Behavioral factors		Patient categories	
		Cases (N = 161)	Control (N = 161)
Death of a family member from any cause	Yes	79 (49.1%)	84 (52.2%)
	No	82 (50.9%)	77 (47.8%)
Think medications for malaria have side effects	Yes	98 (60.9%)	64 (39.8%)
	No	63 (39.1%)	97 (60.2%)
The first place you go when symptoms of malaria appear	Health facilities	114 (70.8%)	157 (97.5%)
	Drug vender	40 (24.8%)	14 (8.7%)
	Traditional healer	35 (21.7%)	33 (20.5%)
	Religious Healer (“Tsebel”, prayer)	19 (11.8%)	19 (11.8%)
	Other	5 (3.1%)	0
Taken traditional medicines when symptoms of malaria appear	Yes	44 (27.3%)	41 (25.5%)
	No	117 (72.7%)	120 (74.5%)
Taken medications by yourself (without a physician's prescription) when symptoms of malaria appear	Yes	46 (28.6%)	38 (23.6%)
	No	115 (71.4%)	123 (76.4%)
Who decides to seek treatment when symptoms of malaria appear in the family?	Father	49 (30.4%)	36 (22.4%)
	Mother	7 (4.3%)	7 (4.3%)
	Father and mother	5 (3.1%)	6 (3.7%)
	The patient	100 (62.1%)	112 (69.6%)
Ever been infected by malaria	Yes	29 (18%)	56 (34.8%)
	No	132 (82%)	105 (65.2%)
Do you drink alcohol	Yes	31 (19.3%)	29 (18%)
	No	130 (80.7%)	132 (82%)
Have you ever been provided any sort of education about malaria	Yes	80 (49.7%)	114 (70.8%)
	No	81 (50.3%)	47 (29.2%)

### Physical and environmental factors affecting treatment-seeking for malaria

**Figure 1:**
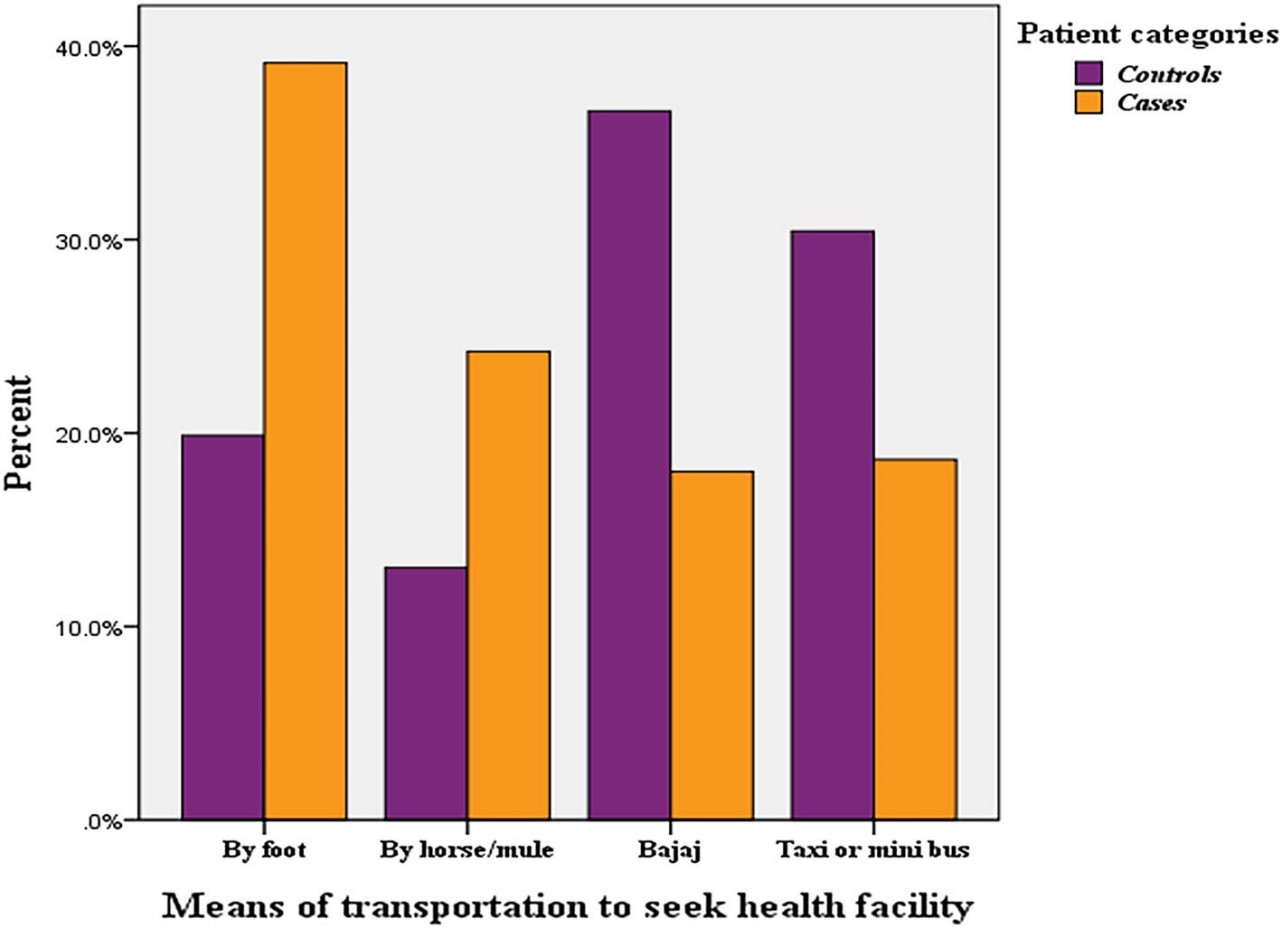
Physical and environmental factors affecting treatment-seeking for malaria among study participants in public health facilities of South Gonder District, 2022(n = 322).

The study revealed that a significant proportion of controls (78.3%, 126 individuals) and a minority of cases (42.2%, 68 individuals) had easy access to transportation. Most cases (39.1%, 63 individuals) relied on walking for transportation to health facilities. Of the controls, 57.1% (92 individuals) and 36% of cases (58 individuals) reached health facilities within 30 min (Fig. 1). The majority of controls (70.2%, 113 individuals) and a few cases (46%, 74 individuals) were covered by community-based health insurance (CBHI).

### Healthcare system-related factors affecting treatment seeking for malaria

The majority of controls (79.5%) and a minority of cases (39.8%) reported a very short waiting period for health services. About one-fourth of controls (24.8%) and nearly half of cases (47.2%) perceived the cost of malaria healthcare as expensive. Most controls (77%) and cases (83.2%) did not encounter shortages in drugs or laboratory services. Three-fourths of controls (78.9%) and a few cases (41.6%) expressed confidence in the health facility services they received (Table 5).

**Table 5:** Health care system related factors affecting treatment seeking for malaria among study participants in public health facilities of South Gonder District, Ethiopia, 2022 (n = 322).

Health care system related factors		Patient categories	
		Cases (N = 161)	Control (N = 161)
How long did it take you to get the service in the health center/hospital	Not long	64 (39.8%)	128 (79.5%)
	Long	97 (60.2%)	33 (20.5%)
How do you think is the health care cost for malaria in the health center/hospital	Expensive	76 (47.2%)	40 (24.8%)
	Cheap	76 (47.2%)	103 (64%)
	No fee	9 (5.6%)	18 (11.2%)
Have you ever experienced a shortage of drugs/laboratory tests in the health center/hospital previously	Yes	27 (16.8%)	37 (23%)
	No	134 (83.2%)	124 (77%)
Are you confident on the malaria care which the health center/hospital is providing currently	Yes	94 (58.4%)	127 (78.9%)
	No	67 (41.6%)	34 (21.1%)

### Determinant factors for delay in treatment seeking for malaria

No Multicollinearity exists among independent variables, as indicated by a Variance Inflation Factor (VIF) of 2.1, well below the threshold of 10.

All variables were entered into the bivariable logistic regression model. Sociodemographic variables, such as sex (p = 0.253), age group (p = 0.087), ethnicity (p = 0.171), marital status (p = 0.818), and religion (p = 0.231) were not crudely associated with delay for treatment-seeking. Other variables such as family death (p = 0.577), and taking traditional medicine (p = 0.705) were not crudely associated with delay in treatment-seeking. The bivariable logistic regression knowledge category was crudely associated with delay for treatment-seeking (COR = 1.92(1.23–2.99). All variables with p-value < 0.2 were entered into the multivariable logistic regression model.

In multivariable logistic regression, educational status, malaria drug side effects, provided health education, transportation accessibility, community-based health insurance, and confidence in malaria care were significantly associated with delays in seeking treatment for malaria.

In this study, Educational status played a crucial role, particularly for those unable to read and write, demonstrating a significantly higher delay compared to other educational categories (AOR = 3.47, p = 0.048). Students also exhibited a distinct trend towards delayed treatment (AOR = 3.34, p = 0.08). Transportation challenges significantly impacted cases, with those facing difficulties almost five times more likely to experience delays (AOR = 4.70, p = 0.02). Lack of community-based health insurance emerged as a significant factor contributing to delay among cases (AOR = 2.50, p = 0.006). Furthermore, cases lacking confidence in malaria treatment had significantly higher odds of delay compared to controls (AOR = 2.14, p = 0.032).

This study revealed that study participants who had no confidence in malaria treatment were 2.14 times more likely to delay seeking treatment for malaria [AOR = 2.14, 95% CI (1.06, 4.29)] with p-value = 0.032 (Table 6).

**Table 6:** Multivariable logistic regression for the determinant of delay in treatment seeking for malaria among study participants in public health facilities of South Gonder District, Ethiopia, 2022(n = 322).

Variables	Delay in treatment-seeking for malaria				
	Cases	Controls	COR (95% CI)	AOR (95% CI)	p-value
Educational status					
Unable to read and write	61	30	4.66(2.43–8.91)	3.47(1.01–11.9)	0.048*
Primary school	48	42	2.61(1.39–4.93)	1.62(0.57–4.59)	0.364
Secondary school	28	34	1.88(0.94–3.77)	0.48(0.16–1.45)	0.19
College and above	24	55	1	1	
Occupation					
Government employee	13	38	0.45(0.17–1.21)	1.76(0.44–6.89)	0.41
Private employee	29	29	1.33(0.53–3.30)	1.68(0.50–5.65)	0.39
Housewife	22	27	1.08(0.42–2.77)	0.83(0.21–3.22)	0.79
Student	38	18	2.81(1.10–7.17)	3.34(0.86–12.9)	0.08
Farmer	34	23	1.97(0.78–4.92)	0.54 (0.14–2.10)	0.37
Daily labourer	13	10	1.73(0.56–5.27)	1.96 (0.46–8.28)	0.35
Merchant	12	16	1	1	
Age group					
< 15 years	18	10	2.18(0.95–4.98)	1.71(0.47–6.22)	0.41
15–29 years	59	49	1.46 (0.90–2.35)	1.19(0.59–2.41)	0.61
> 30 years	84	102	1	1	
Knowledge category					
Inadequate knowledge	90	64	1.92(1.23–2.99)	0.58(0.25–1.32)	0.19
Adequate knowledge	71	97	1	1	
Malaria drug has the side effect					
Yes	98	64	2.35(1.50–3.68)	1.89(1.04–3.42)	0.03*
No	63	97	1	1	
Infected by malaria					
Yes	29	56	0.41(0.24–0.69)	0.86(0.42–1.76)	0.68
No	132	105	1	1	
Provided health education on malaria					
Yes	80	114	1	1	
No	81	47	2.45(1.55–3.88)	1.93(1.02–3.65)	0.04*
Transportation accessibility					
Yes	68	126	1	1	
No	93	35	4.92(3.02–8.02)	4.70(1.73–12.7)	0.02*
Means of transportation					
By foot	63	32	3.21(1.72–5.99)	0.74(0.24–2.32)	0.61
By horse or mule	39	21	3.03(1.50–6.09)	1.58(0.47–5.24)	0.45
Bajaj	29	59	0.80 (0.42–1.51)	0.89(0.39–2.02)	0.78
Taxi or minibus	30	49	1	1	
Members of community-based health insurance					
Yes	74	113	1	1	
No	87	48	2.76(1.75–4.37)	2.50(1.30–4.82)	0.006*
Confidence in malaria treatment					
Yes	94	127	1	1	
No	67	34	2.66 (1.62–4.35)	2.14(1.06–4.29)	0.032*

*Significantly associated variables.

## Discussion

The World Health Organization (WHO) advocates swift access to suitable anti-malarial treatment, preferably within the initial 24 h of fever onset, to prevent progression from uncomplicated to severe malaria^7^. Key findings indicate that unable to read and write, fear of malaria drug side effects, lack of health education, limited transportation, absence of community-based health insurance, and lack of confidence in health facility care contribute to treatment-seeking delays among malaria patients. This study revealed that delay in treatment-seeking among malaria patients had a significant association with educational status, malaria drug side effects, getting health education on malaria, transportation access, CBHI membership, and confidence in health malaria care. The current findings were similar to other studies conducted in North West Ethiopia that showed community-based health insurance, previous history of malaria infection, and distance were determinants of delay in seeking treatment for malaria^9^.

The current study showed that the educational status of the patient was found to be an important determinant factor for treatment-seeking delay among malaria patients. In this study patients who were unable to read and write were more likely to delay seeking treatment for malaria. This finding was comparable with the study conducted in Myanmar that showed patients who were unable to read and write were more likely to delay seeking treatment for malaria^26^, and a similar finding was reported in a study conducted in the Tigray region^17^. The relationship between educational level and early health-seeking might be because literate people are better able to understand health education messages delivered by different mass media and health professionals. Develop materials that can be understood if the existing health education materials are not comprehended by people with no/poor literacy—then need.

A side-effect of anti-malarial drugs was also found to be a significant factor for delays in treatment-seeking among malaria patients in health facilities. Study participants who experienced side effects of anti-malarial drugs were more likely to delay seeking treatment when they needed malaria treatment. This association might be due to the reason that most of the patients did not visit healthcare early due to fear of its side effects. A consistent finding was reported in other studies conducted in Southern Ethiopia 2.91 times and Southwest Ethiopia 4.96 times more likely to be late for the treatment of malaria care^15,20^. These findings contradict a study conducted in the Northwestern Zone of the Tigray region that showed side effects of anti-malarial drugs had no significant association with delay in treatment-seeking^17^. They may prioritize avoiding potential discomfort from medication over seeking immediate treatment for their illness.

In this study, study participants who had not received any health education regarding malaria were more likely to delay treatment-seeking for malaria. This could be due to those who did not receive health education on malaria disease as a very simple disease and also understood there was no treatment at health facilities due to a lack of knowledge on the treatment. On the other hand, individuals who had received adequate health education on malaria were found to be highly motivated to visit health facilities as early as possible due to sufficient information regarding all of the side effects and their severity. This finding was also concomitant with other study findings from North-western Ethiopia^27^.

The current study showed that study participants, who had no transportation access were more likely to be late in seeking treatment for malaria. This finding was comparable with other studies conducted in Uganda that showed that long distance from the place of residence to public health facilities as a result of transportation access was a notable risk factor for both delayed care seeking and severe malaria^28^ and a study from North West Ethiopia was also revealed study participants who traveled for more than 30 min to get to a health facility due to lack of transportation access were more likely to be late in seeking treatment for malaria^9^. The possible explanation for the finding is that study participants who had no transportation access may have faced difficulties in reaching healthcare facilities promptly. This could be due to limited public transportation options, lack of personal vehicles, or living in remote areas with poor road infrastructure**.** In addition***,*** community-based diagnosis and treatment be considered using community malaria volunteers or community volunteer health volunteers.

This study revealed that members of the CBHI were an important determinant of delayed treatment seeking. Study participants responded that no member of CBHI was likely to delay seeking treatment for malaria. The possible explanation for this could be the shortage of money for a fee for malaria treatment. This finding was in line with a study conducted in Ghana has concluded that those who were members of health insurance schemes were less likely to delay anti-malarial treatment seeking by seeking traditional/herbal medicine and self-medication^31^. Furthermore, a study conducted in Ethiopia has revealed that patients who were not members of community-based health insurance (CBHI) were more likely to delay in seeking treatment when compared to those who were members of CBHI^9^. Confidence in the malaria care provided by health facilities was also another determinant factor of delay in treatment-seeking among malaria patients. This study revealed that study participants who had no confidence in malaria care were more likely to be late to seek treatment. The study's limitations include potential recall bias in participant responses, generalizability constraints to other settings, and reliance on self-reported data, which may introduce subjectivity.

## Conclusion

In Summary, this study revealed significant associations between delays in seeking malaria treatment and factors such as educational status (those who were unable to read and write), malaria drug side effects, health education on malaria, transportation access, CBHI membership, and confidence in health malaria care. It is recommended that targeted interventions and awareness campaigns be implemented to address these determinants, promoting prompt and effective malaria treatment-seeking behavior in the studied population (Supplementary Information).

### Supplementary Information

Supplementary Information.

## Data Availability

All the necessary data are included in the manuscript. An English version data collection tool and detailed operational definitions of the outcome variable are accessible at a reasonable request from the corresponding author**.**

## Abbreviations

AIDS: Acquired immune deficiency virus
BF: Blood film
CBHI: Community based health insurance
CI: Confidence interval
ETB: Ethiopian birr
FMOH: Federal Ministry of Health
HIV: Human immune virus
MDG: Millennium development goal
NMCP: National Malaria Control Programme
PMI: United States, President's malaria initiative
RBM: Roll back malaria
RDT: Rapid diagnostic test
USD: United States dollar
WHO: World Health Organization
SPSS: Statistical Package for Social Sciences
AOR: Adjusted odds ratio
