# Supplementary material for: Factors influencing delay in malaria treatment seeking at selected public health facilities in South Gonder, Ethiopia
Source: Sci Rep. 2024 Mar 20;14:6648. doi: 10.1038/s41598-024-56413-7 (PMC10951229; doi:10.1038/s41598-024-56413-7)
Supplement: Supplementary file 1 — Supplementary Information. [file 41598_2024_56413_MOESM1_ESM.docx]

**Part 1: patients’ socio-economic and demographic characteristics**

| Q no | Questions | Options/responses | Skip |
| --- | --- | --- | --- |
| 101 | Residence | 1. Urban 2. Rural |  |
| 102 | Age | ________________ |  |
| 103 | Sex | 1. Male 2. Female |  |
| 104 | Ethnicity | 1.Amhara  99.other_____________ |  |
| 105 | Your current marital status | 1. Single 2. Married 3. Widowed 4. Divorced/Separated |  |
| 106 | To which religion do you belong? | 1. Orthodox Christian 2. Muslim   3.Other specify____________ |  |
| 107 | What is your highest level of education Completed? | Never attended formal school/Illiterate   1. Primary school(1-8) 2. Secondary school(9-12) 3. College and above   5.Other(specify)_______________ |  |
| 108 | What is your main occupation? | 1. Government employee 2. Private employee 3. House wife 4. Student 5. Farmer 6. Daily laborer 7. Merchant |  |
| 109 | Your family size | _______________(in number) |  |
| 110 | Monthly income | _______________ETBirr |  |

**Part 2: Timeliness treatment seeking for malaria**

| S.no | Questions | Options/responses | Skip |
| --- | --- | --- | --- |
| 201 | How long is it since the onset of the first malaria symptom? | 1. <24 hours 2. 24-48 hours 3. 48-72 hours 4. >3 days |  |
| 202 | If you came to health  facility after 24 hours of  the onset of symptoms  What was your main reason? | 1.Disease was not sever  2.Cost of medical care  3.No access of transportation  4.Treating at home  5.Buy medication from drug vendor  6.Taking traditional medicine |  |
| 203 | Source of information about malaria | 1.TV/radio/newspaper  2.Health worker  3.Friends/neighbor  4.health facility  5.Other__________ |  |

**Part 3: Malaria knowledge assessment questions**

| Q no | Questions | Options/responses | Skip |
| --- | --- | --- | --- |
| 301 | Have you ever heard about malaria? | 1.Yes  2.No |  |
| 302 | Is malaria caused by mosquito bite? | 1.Yes  2.No |  |
| 303 | Can malaria be prevented? | 1.Yes  2.No |  |
| 304 | If your answer for Q303 is „Yes‟, how it can be prevented? | 1.Environmental management  2.By using insecticide treated nets  3.Indoor residual spray  4.Prophylaxis  5.Don‟t know |  |
| 305 | Can malaria be cured? | 1.Yes  2.No |  |
| 306 | Can malaria cause death? | 1.Yes  2.No |  |
| 307 | Which symptoms of malaria do you know? | 1.Fever  2.Headache  3.Body pains and fatigue  4.Shivering/chills  5.Loss of appetite  6.Nausea/vomiting  7.Sweating  8.Don‟t know |  |
| 308 | When does a mosquito bite? | 1.Day time  2. Night  3. Always  4. I do not know |  |
| 309 | Do you know the resting places of mosquitoes? | 1.Yes  2.No |  |
| 310 | Do you know the breeding sites mosquitoes? | 1.Yes  2.No |  |
| 311 | If „ Yes‟ for question 310 where? | 1.Stagnant water  2.Bushes/grass   1. Large tree cavities 2. Trash cans/Garbage’s 3. 5.Don‟t know |  |
| 312 | Do you know how to prevent mosquitoes breeding? | 1.Yes  2.No |  |
| 313 | If „Yes‟ for Q 312, how? | 1.cleaning household surrounding  2.Dainage of stagnant water  3.Clearing bushes/grass  4.Don‟t know |  |

**Part 4:- Questions on Behavioral factors**

| Q no | Questions | Options/responses | Skip |
| --- | --- | --- | --- |
| 401 | Is there a death of a family member from any cause? | 1. Yes 2. No |  |
| 402 | Do you think that medications for malaria have side effects? | 1.Yes  2.No |  |
| 403 | Where is the first place you go when symptoms of malaria appear? | 1.Health facilities  2.Drug vender  3.Traditonal healer 4.Religious  healing(tsebel, prayer)  5.Other |  |
| 404 | Do you take traditional medicines when symptoms of malaria appear? | 1.Yes  2.No |  |
| 405 | Do you take medications by yourself (without physician prescription) when symptoms of malaria appear? | 1.Yes  2.No |  |
| 406 | Who decides to seek treatment when symptoms of malaria appear in the family? | 1.Father  2.Mother  3.Father and mother  4.The patient |  |
| 407 | Have you ever been infected by malaria? | 1.Yes  2.No |  |
| 408 | If “yes” for Q407 how often? | 1.Sometimes  2.Frequently |  |
| 409 | Do you smoke? | 1.Yes  2.No |  |
| 410 | If “Yes” for Q409, how much packs per day? | 1.<1 pack  2.1 pack  3.2 packs  4.>2 packs |  |
| 411 | Do drink alcohol? | 1.Yes  2.No |  |
| 412 | If “Yes” for Q411, how often? | 1.Some times  2.Frequently |  |
| 413 | Do you chew khat? | 1.Yes  2.No |  |
| 414 | If “Yes” for Q413, how often? | 1.Sometimes  2.Frequently |  |
| 415 | Have ever been provided any sort of education about malaria? | 1.Yes  2.No |  |

**Part 5:-Questions on physical and environmental factors**

| Q no | Questions | Options/responses | Skip |
| --- | --- | --- | --- |
| 501 | How long it takes you from your home to reach the health center/hospital? | _______________minutes |  |
| 502 | Is transportation easily accessible? | 1.Yes  2.No |  |
| 503 | Which means of transportation do you use when you want to come to health facility? | 1.On foot  2.On horse/mule  3.Motor bicycle/Bajaj  4.Car |  |
| 504 | How do you think the cost transportation? | 1.Expensive  2.Cheap  4.No fee |  |
| 505 | Are you a member of CBHI? | 1.Yes  2.No |  |

**Part 6 :- Questions on health facility related factors**

| Q no | Questions | Options/responses | Skip |  |
| --- | --- | --- | --- | --- |
| 601 | How long did it take you to get the service in the health center/hospital? | 1.Not long  2.long |  |  |
| 602 | How do you think is the health care cost for malaria in the health center/hospital? | 1.Expensive  2.Cheap  4.No fee |  |  |
| 603 | Have you ever experienced a shortage of drugs/laboratory tests in the health center/hospital previously? | 1.Yes  2.No |  |  |
| 604 | Are you confident on the malaria care which the health center/hospital is providing currently? | 1.Yes  2.No |  |  |
